# Questions over high frequency of mutant PfATP6 haplotypes in traveller isolates

**DOI:** 10.1186/1475-2875-11-186

**Published:** 2012-06-08

**Authors:** Charles J Woodrow, Kate B Gardner, Leyla Y Bustamante

**Affiliations:** 1 Mahidol-Oxford University Tropical Medicine Research Unit (MORU), Bangkok, Thailand; 2 Sanger Malaria Programme, Wellcome Trust Sanger Institute, Hinxton, Cambridge, UK

## Abstract

A recent paper in Malaria Journal suggests that a high proportion of *Plasmodium falciparum* isolates found in travellers returning from a range of African countries carry the PfATP6 A623E S769N haplotype, and that this genotype is associated with artemether resistance. Such a finding would represent a substantial departure from the extensive literature reporting these individual mutations to be very rare, with the double mutation never documented. The number of isolates screened to obtain these double mutants is unstated, but highly relevant, not least because selection of isolates could have introduced significant confounders, such as timing of in vitro testing. An additional concern relates to the location of sequencing primers used to assess these positions. In the absence of clear information on these fundamental questions it would be appropriate to treat the findings with caution.

## 

Pillai *et al.*[1] describe a possible association between the PfATP6 A623E S769N haplotype and artemether resistance in *Plasmodium falciparum* isolates in returning travellers from eight African countries. A large number of studies have been undertaken specifically to look for these and other polymorphisms at a global level, both before and after the introduction of artemisinin combination therapy. As the authors point out, A623E and S769N have never been reported in the same isolate, and even as separate entities they have only been found at very low prevalence in a small number of countries. Among approximately 1,000 samples studied originating in the eight countries described in the report, only two isolates have been found with the A623E mutation (in Tanzania) and no isolate has been found with the S769N mutation [2-10] (Table 1).

**Table 1:** **Previous publications on prevalence of the A623E and S769N polymorphisms in the countries described by Pillai *et al.***[1]

	**A623E**	**Reference**	**S769N**	**Reference**
Nigeria	0/11	[3]	0/11	[3]
Ghana	0/120	[3] (0/13), [4] (0/1), [7] (0/38), [9] (0/68)	0/120	[3] (0/13), [4] (0/1), [7] (0/38), [9] (0/68)
Angola	0/76	[3] (0/10), [6] (0/66)	0/76	[3] (0/10), [6] (0/66)
Cameroon	0/160	[3] (0/2), [5] (0/97), [8] 0/61	0/160	[3] (0/2), [5] (0/97), [8] 0/61
Tanzania	2/636	[2] (0/288), [4] 2/279, [7] 0/69,	0/635	[2] (0/197), [4] 0/279, [7] 0/69, [10] 0/90
Liberia	0/1	[4]	0/1	[4]
Kenya	0/4	[3]	0/4	[3]
Total	2/1008		0/1007	

The data indicate numbers of samples with mutation/number of samples assayed. In cases where the precise number of samples examined for each SNP was not reported the lowest possible number within the range described is shown.

The finding of A623E S769N double mutants in 11 of 28 patients in one centre is, therefore, highly unexpected. The authors propose that natural selection for these polymorphisms may be taking place in the countries of origin for these parasites, because of the rapid scale up of anti-malarial treatment programmes being implemented in recent years. However, before natural selection is invoked, a much more basic concern needs to be addressed, namely selection of isolates in the laboratory. It is notable that the median date of receipt for the mutant isolates was around 500 days before that of the wild-type samples (according to the supplementary data). This is of profound methodological importance since use of wild-type controls with different properties to the mutant population (such as date of assay) would introduce a significant confounder. How many isolates were screened to obtain the final 11 double mutants? And how were the wild-type controls selected?

Another possible explanation for the high reported frequency of mutant haplotypes relates to the methods used to sequence the PfATP6 gene itself. Examination of the sequencing primers appears to indicate that the forward biotinylated primer reported by the authors to have been used to assess the A623E polymorphism lies over the A623 position itself (Figure 1). Since primer is incorporated into product, it is difficult to understand how position 623 was assessed, and specifically how A623E mutants could have been seen at all.

**Figure 1:**
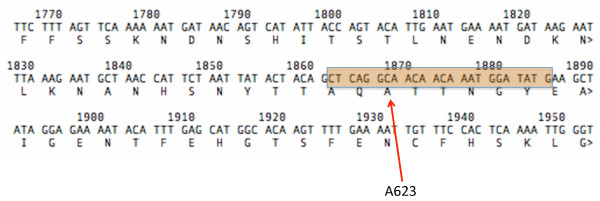
**Location of the forward biotinylated primer used to assess the A623E polymorphism in the PfATP6 coding sequence.** The primer lies over the A623 position itself.

In the absence of clear information on these fundamental questions it would be appropriate to treat the findings and conclusions of the article with caution.

## Competing interests

The authors declare that they have no competing interests.

## Authors' contributions

All authors contributed to meta-analysis of publications. CW drafted the text. All authors read and approved the final manuscript. 
